# TLR2/4 are novel activating receptors for SARS-CoV-2 spike protein on NK cells

**DOI:** 10.3389/fimmu.2024.1368946

**Published:** 2024-05-31

**Authors:** Nadine Landolina, Biancamaria Ricci, Irene Veneziani, Claudia Alicata, Francesca Romana Mariotti, Andrea Pelosi, Linda Quatrini, Eva Piano Mortari, Rita Carsetti, Paola Vacca, Nicola Tumino, Bruno Azzarone, Lorenzo Moretta, Enrico Maggi

**Affiliations:** ^1^ Tumor Immunology Unit, Bambino Gesù Children’s Hospital, IRCCS, Rome, Italy; ^2^ Innate Lymphoid Cells Unit, Immunology Research Area, Bambino Gesù Children’s Hospital, IRCCS, Rome, Italy; ^3^ B cell Unit, Immunology Research Area, Bambino Gesù Children’s Hospital, IRCCS, Rome, Italy

**Keywords:** NK-cell, SARS-CoV2, spike glycoprotein, TLRs, variants of concerns

## Abstract

**Background:**

In early infected or severe coronavirus disease 2019 (COVID-19) patients, circulating NK cells are consistently reduced, despite being highly activated or exhausted. The aim of this paper was to establish whether severe acute respiratory syndrome coronavirus 2 (SARS-CoV-2) spike glycoprotein (SP) may directly trigger NK cells and through which receptor(s).

**Methods:**

SP-stimulated human NK cells have been evaluated for the expression of activation markers, cytokine release, and cytotoxic activity, as well as for gene expression profiles and NF-kB phosphorylation, and they have been silenced with specific small interfering RNAs.

**Results:**

SPs from the Wuhan strain and other variants of concern (VOCs) directly bind and stimulate purified NK cells by increasing activation marker expression, cytokine release, and cytolytic activity, prevalently in the CD56^bright^NK cell subset. VOC-SPs differ in their ability to activate NK cells, G614, and Delta-Plus strains providing the strongest activity in the majority of donors. While VOC-SPs do not trigger ACE2, which is not expressed on NK cells, or other activating receptors, they directly and variably bind to both Toll-like receptor 2 (TLR2) and TLR4. Moreover, SP-driven NK cell functions are inhibited upon masking such receptors or silencing the relative genes. Lastly, VOC-SPs upregulate CD56^dim^NK cell functions in COVID-19 recovered, but not in non-infected, individuals.

**Conclusions:**

TLR2 and TLR4 are novel activating receptors for SP in NK cells, suggesting a new role of these cells in orchestrating the pathophysiology of SARS-CoV-2 infection. The pathogenic relevance of this finding is highlighted by the fact that free SP providing NK cell activation is frequently detected in a SARS-CoV-2 inflamed environment and in plasma of infected and long-COVID‐19 subjects.

## Introduction

The coronavirus disease 2019 (COVID-19) pandemic due to severe acute respiratory syndrome coronavirus 2 (SARS-CoV-2) started in 2019 in Wuhan (China) and caused almost 7.5 million documented deaths until 2023. Although originally defined as a respiratory viral infection, COVID-19 is now clearly recognized as a more complex, multistep, multi-organ, and immune-mediated disease. Among the alterations of innate immunity induced by SARS-CoV-2 infection, the dysfunction of circulating NK cells in early infected or severe patients has been repeatedly reported (1, 2). The absolute number of NK cells is reduced along the infection (3, 4) and is restored after recovery (5, 6). By contrast, people undergoing a fatal outcome displayed a progressive loss of these cells evident already at the onset of the symptoms (7). The two main human NK cell subsets (CD56^bright^CD16− and CD56^dim^CD16+) have been rarely investigated in COVID-19, while the study is highly informative in view of their different distribution and function (8, 9). An inverse correlation between the number of NK cells and viral load has been documented, suggesting that the number of circulating NK cells might represent a prognostic parameter to predict the outcome of the disease (10).

Despite their reduction, NK cells display a strong activation and proliferation in both peripheral blood (PB) and bronco-alveolar lavage fluid (BALF) from COVID-19 patients, as shown by the expression CD69 and Ki-67 (11). Notably, at the disease onset, patients who will develop severe COVID-19 usually show increased plasma levels of pro-inflammatory cytokines, including IFN-γ mostly released from activated NK cells (12). The peculiar phenotype of NK cells is also characterized by the increased expression of checkpoint inhibitors (LAG3, PD-1, and TIM-3) or inhibitory receptors (NKG2A/CD94) mainly in the CD56^dim^ subset, suggesting a dysfunctional/exhausted profile (13–15). Such phenotype, which is mainly observed in patients with severe disease, suggests that NK cells may favor the pathogenesis rather than fight infection (16).

Notably, viral components could play some role in the pathogenesis of COVID-19; circulating Spike protein (SP) and virus RNA have been found in patients with early disease (when humoral immune response is not present yet), post‐acute sequelae of COVID‐19 (PASC) or post-RNA vaccination, which could possibly be responsible for neuro-PASC and post-vaccination myocarditis (17–19). The plasma levels of SP and/or viral RNA were increased in the PASC-positive patients as in the acute phase of COVID-19 (20). The great majority of viruses have evolved mechanisms to interact with NK cells [from the specific binding to entry receptors to non-specific links to several surface molecules, including Toll-like receptors (TLRs)] ultimately favoring cell infection (21–24).

The present study aimed to assess whether SP may exert a direct effect on NK cells and play a role in their hyperactivation in COVID-19.

We show that recombinant SP (rSP) from the SARS-CoV-2 Wuhan strain or other variants of concern (VOCs) directly bind to and stimulate NK cells, resulting in upregulation of activation markers, cytokine production, and cytotoxicity. Remarkably, VOC-rSPs do not trigger ACE2 and activating receptors on NK cells, but rather directly bind TLR2 and TLR4 and mediate activation of NK cells, which were inhibited by masking such receptors. In addition, phosphorylation of NF-kB and release of IFN-γ are downregulated in rSP-stimulated NK cells silenced for TLR2 and TLR4 genes. Lastly, VOC-rSPs induce high CD56^dim^ NK cell activation and IFN-γ release in recovered, but not in not infected, individuals. These findings indicate TLR2 and TLR4 as novel molecular targets for spike viral protein and may re-design a novel role for NK cells in the pathophysiology of COVID-19.

## Materials and methods

### Reagents

All reagents used in this paper and their source are listed in **Supplementary Table 1**.

### Spike glycoprotein from ancestral and variants of concern

rSPs from different VOC derive from the following lineages: Ancestral Wuhan strain (lineage A), Alpha or G614 (lineage B.1.1.7), Indian Delta-Plus (lineages B.1.617.2.1 with mutation K417N), and Omicron (lineage B.1.1.529.2). For simplicity, Wuhan, G614, Delta-Plus, and Omicron were throughout the article.

### Human samples

PB was obtained from buffy coat of healthy donors (HD) admitted to the blood transfusion service of Bambino Gesù Children’s Hospital, Rome, Italy, after obtaining informed consent. The ethical committee approved the study that was conducted according to the Declaration of Helsinki tenets (2058_OPBG_2020). In this study, we also used peripheral blood mononuclear cells (PBMCs) from 11 informed subjects who showed recent (more than 3 months) mild infection/recovery (clinically and antigenically documented) from COVID-19 (Covid+) and from six informed individuals with no clinical and serological signs (absence of anti-nucleocapsid antibodies) of the disease (Covid−). At the sampling time, Covid+ subjects were antigen-negative. All selected individuals had received at least three doses of Comirnaty mRNA vaccine Pfizer/BioNTech (New York, USA), were negative for HBV, HCV, and HIV infections; and were cancer free.

### PBMC and NK cell isolation

Human NK cells (>98% CD3^−^CD56^+^) were isolated from PBMCs as previously described (25, 26). Briefly, following PBMC isolation by density Ficoll-Paque Plus, cells were resuspended in RosetteSep NK-cell enrichment cocktail (STEMCELL Technologies cat #15065) and NK cells were negatively selected by density centrifugation. Fresh NK cells were cultured in RPMI-1640 medium supplemented with 2 mM L-glutamine (Euroclone), 1% penicillin and streptomycin (Euroclone), and 10% fetal bovine serum (FBS; Thermo-Fisher Scientific, Massachusetts, USA). For masking experiments, NK cells were incubated with neutralizing antibodies (2 µg/mL, see **Supplementary Table 1**), in culture medium for 1 h followed by further stimulation with VOC-rSPs for 72 h at 37°C. At the end of culture, cell pellets were harvested and supernatants were collected for further analysis.

In order to exclude myeloid contaminants in NK cell preparations, evaluation of CXCL10, a typical cytokine produced by myeloid cells, was performed in the culture supernatants by ELISA.

### Cell lines, culture conditions, and masking experiments

The NALM-18 (B-cell childhood acute lymphoblastic leukemia) cell line was kindly provided by Dr. Pende D (IRCCS, Policlinico San Martino, Genoa, Italy), while HEK-293 (human embryonic kidney) Blue Null1, TLR2, and TLR4 reporter cell lines were purchased from InvivoGen (San Diego, California, USA). These cell lines were grown in RPMI-1640 (Euroclone, Milan, Italy) and DMEM, respectively, supplemented with 2 mM L-glutamine (Euroclone), 1% penicillin and streptomycin (Euroclone), and 10% FBS (Thermo-Fisher Scientific, Massachusetts, USA). HEK-293 Blue Null expression and TLR2 and TLR4 expression on reporter cell lines were maintained according to manufacturer’s specifications.

### Spike binding

Freshly isolated NK cells were incubated with biotinylated spike protein (2.5 µg/mL Biotechne, Minneapolis, USA) and streptavidin PE [Becton Dickinson (BD), New Jersey, USA] in PBS at 4°C for 1 h. This mix was previously kept at 4°C for 1 h. Afterwards, cells were stained with fluorescent-labeled antibodies for flow cytometry detection as specified in **Supplementary Table 1** (Reagent list).

### Flow cytometry

Surface staining and intracellular staining of VOC-rSP-stimulated NK cells were performed with fluorescent-labeled antibodies to membrane molecules or cytokines, as previously described (25). Evaluation of apoptosis was carried out with FITC-conjugated Annexin V (BD, New Jersey, USA) and propidium iodide (PI; Sigma-Aldrich, Darmstadt, Germany) as previously described (26). Analysis of specific staining was performed by Cytoflex LX flow cytometer cytexpert 2.4 (Beckman Coulter, Brea, California.) and FlowJo Software (BD) and Kaluza software (Beckman Coulter).

### NK cell cytotoxic assay, CD107a expression, and cytokine assessment

NK cell cytotoxic activity was evaluated by a flow cytometric assay by using NALM-18 cells as targets, as previously described (25, 27). For CD107a expression, either untreated or Wuhan/G614-treated NK cells (72 h) were co-cultured for 4 h with NALM-18 target cells, the effector:target ratio being 1:1. Anti-CD107a eFluor 660 (Invitrogen) and GolgiStop (1:500) were added during stimulation. Afterwards, cells were stained with fluorescent-labeled antibodies for flow cytometry detection as specified in **Supplementary Table 1** (Reagent list). For intracellular staining, cells were permeabilized using FOXP3 Staining buffer set (Miltenyi Biotec).

The presence of IFN-γ, TNF-α, and CXCL10 was investigated in the supernatants from NK cell cultures by DuoSet ELISA kits (R&D Systems Inc, Minneapolis, USA) according to the manufacturer’s instructions (Reagent list, **Supplementary Table 1**) The cutoff limits of the assays were as follows: 18.76 pg/mL for IFN-γ, 15.6 pg/mL for TNF-α, and 31.2 pg/mL for CXCL10.

### TLR2/TLR4 reporter assay

Specific stimulation of human TLRs was carried out in HEK-293 Blue Null1, TLR2, and TLR4 reporter cells by measuring the activation of NF-κB (InvivoGen) (28), according to the manufacturer’s instructions.

### Transfections

TLR2 and TLR4 silencing experiments on freshly isolated NK cells were performed as previously described with minor changes (27). TLR2, TLR4, and negative control small interfering RNA (siRNA) are reported in **Supplementary Table 1**. Following 48-h incubation, cells were activated with Wuhan-rSP (2.5 µg/mL) for 24 h in culture medium. Following this, cell pellets were processed for Western blot analysis whereas supernatants were collected for further analysis.

### RNA extraction, cDNA synthesis, and PCR analysis

Total RNA extraction, reverse transcription, and gene expression studies on bulk, untreated, Wuhan- and G614-rSP-stimulated (6h) or transfected freshly purified NK cells were performed as previously described (25, 27). Total RNA extraction from NK cells was performed with an RNeasy Plus mini kit (Qiagen GmbH, cat #74134) following the manufacturer’s protocols. RNA was reverse-transcribed with oligo-dT primers using the Super Script IV first-strand synthesis (Invitrogen, cat #18091050) system following the manufacturer’s instructions. Reactions were carried out on a QuantStudio 7 Flex instrument using the recommended PCR cycling conditions. The following primers were used for TLR-2: forward 5′-CTT CAC TCA GGA GCA GCA AGC A-3′, reverse 5′-ACA CCA GTG CTG TCC TGT GAC A-3′; TLR-4: forward 5′-TGG ACC TGA GCT TTA ATC CC-3′, reverse 5′-GTC TGG ATT TCA CAC CTG GAT-3′, GAPDH: forward 5′-TCTTTTGCGTCGCCAGCCGA-3′, reverse 5′-ACCAGGCGCCCAATACGACC-3′.

PCR array was performed in custom 384-well TaqMan array microfluidic cards (Thermo Fisher Scientific, cat #4342259) to detect selected genes implicated in NK cell biology (ID reported in **Supplementary Table 2**). Analysis of PCR array data was performed by using relative threshold algorithm and normalized using the mean of β-actin expression, used as reference gene (TaqMan assay ID Hs99999903_m1, Applied Biosystems and Thermo-Fisher Scientific).

### Western blotting

Whole-cell extracts from untreated, mock-, or TLR2/TLR4-silenced NK cells were examined to detect TLR2 and TLR4 and phosphorylated/total NF-kB, respectively, as previously described (29).

### Statistical analysis

Data were analyzed by GraphPad Prism V.9.3.1 (San Diego, California). Reported values have been plotted as means ± SEM of independent experiments. The exact number of each experiment is reported in the figure legends. If not otherwise specified in the legend, statistical analysis was performed using paired Student’s *t*-test with Bonferroni adjustment and ANOVA Kruskal–Wallis multiple comparison test.

## Results

### VOC-rSPs activate resting NK cells and improve their function

**Figure 1:**
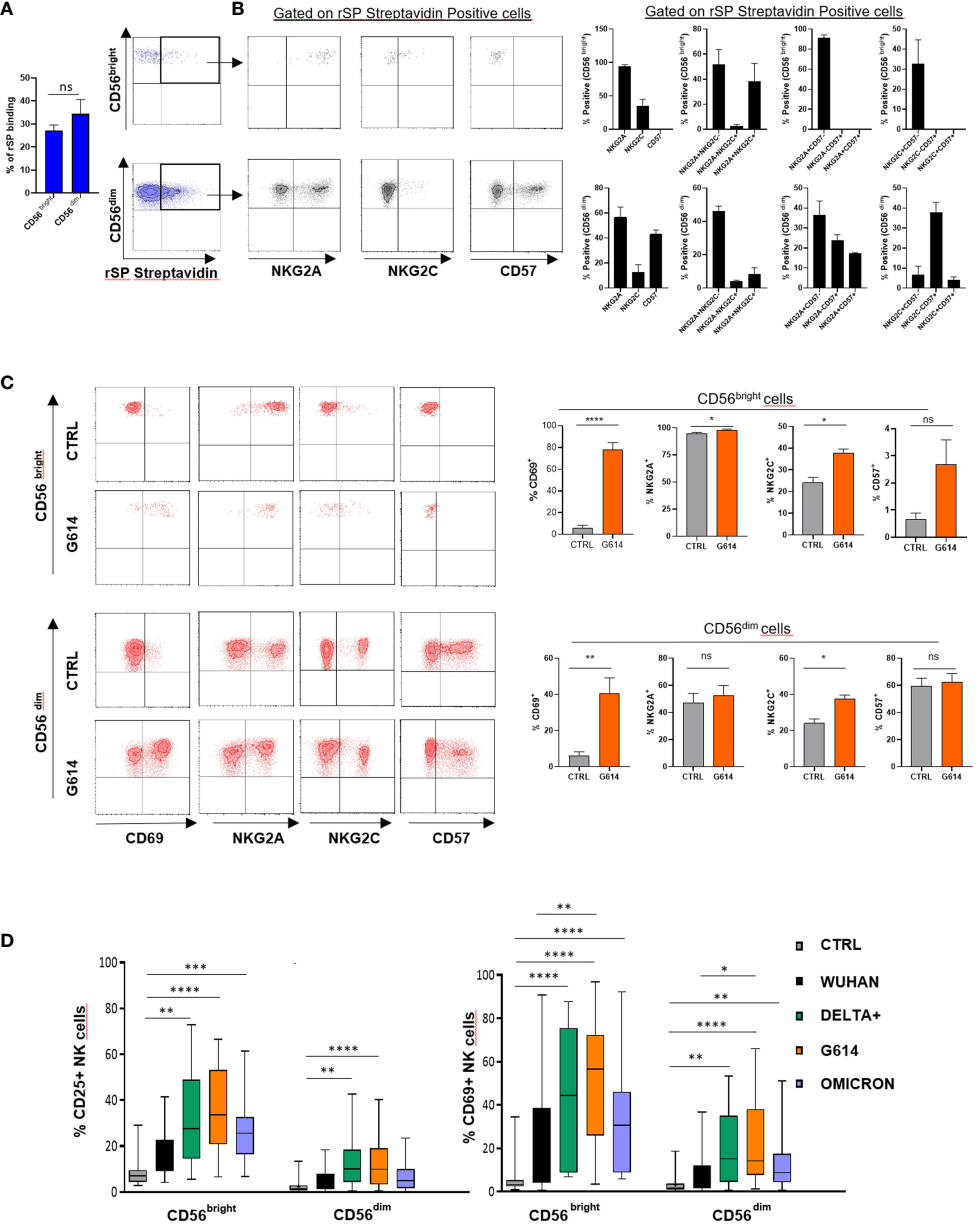
rSP binds and activates NK cells. **(A)** Mean values ± SEM and representative dot plots of binding of biotinylated rSP (Wuhan) to freshly isolated NK cell subsets. **(B)** Expression of NKG2A, NKG2C, and CD57 on rSP-streptavidin positive CD56^bright^ and CD56^dim^ NK cell subsets. One representative dot plot (left panels) and the mean values ± SEM of three independent experiments (right panels) are reported. **(C)** Expression of CD69, NKG2A, NKG2C, and CD57 on untreated (CTRL) and rSP (G614)-stimulated CD56^bright^ and CD56^dim^ NK cells. Data of one representative experiment (left panels) and the mean values ± SEM of seven experiments (right panels) are reported. Statistical analysis was performed using a paired *t*-test. **(D)** CD25 and CD69 expression on the CD56^bright^ and CD56^dim^ NK cell subsets stimulated with different VOCs. Data are plotted as percent of expression on at least 20 HD. Statistical analysis was performed using paired ANOVA Kruskal–Wallis multiple comparison test *p < 0.05; **p < 0.01; ***p < 0.001; ****p < 0.0001. ns: not significant.

To establish whether rSP can directly interact with NK cells, we evaluated the binding of biotinylated rSP (Wuhan strain) on freshly purified NK cells. Even though a high variability was observed, biotinylated rSP bound the surface of a similar proportion of CD56^bright^ and CD56^dim^ NK cells (**Figure 1A**), mostly associated with NKG2A and NKG2C expression on the former and NKG2A and CD57 on the latter subset (**Figure 1B**).

To establish whether rSP could stimulate NK cell function and also to optimize the experimental conditions, we assessed the activation (CD25) and apoptosis (Annexin V) markers’ expression on NK cell-gated (CD3^-^CD56^+^) rSP-stimulated PBMCs from HD, enrolled irrespective of their previous infection and/or vaccination. The time-course experiments indicated that, upon Wuhan-rSP stimulation, the proportion of activated NK cells was significantly increased, peaking at 72 h (**Supplementary Figure 1A**), with the optimal rSP dose established as 2.5 μg/mL (**Supplementary Figure 1A**). Such conditions were used in all experiments, unless otherwise specified. In addition, Annexin V expression was not changed upon the same experimental conditions (**Supplementary Figure 1B**), thereby suggesting that apoptosis was poorly affected by rSP (**Supplementary Figure 1B**).

In order to assess whether rSP exerted direct activity on the NK cell function, purified (>98%) NK cells obtained from PBMCs of HD were stimulated with rSP from different VOCs (Wuhan, G614, Delta-plus, and Omicron). Activated cells were then analyzed for phenotype, activation marker expression, cytokine release, and cytotoxic activity.

rSP (G614) stimulation did not modify the CD57 and NKG2A expression on NK cells, whereas it slightly, but significantly, increased that of NKG2C, without, however, changing the proportions of different NK cell subsets (**Figure 1C**). In the presence of all VOCs, both CD56^bright^ and CD56^dim^ NK cells displayed higher levels of surface CD69 than controls (**Supplementary Figure 1C**). Notably, the recombinant S1 subunit, more than the S2 subunit, was highly effective in inducing NK cell activation (**Supplementary Figure 2A**), whereas, among SARS-CoV-2 glycoproteins (gps), only E gp slightly increased CD25 expression on CD56^bright^ NK cells (**Supplementary Figure 2B**).

**Figure 2:**
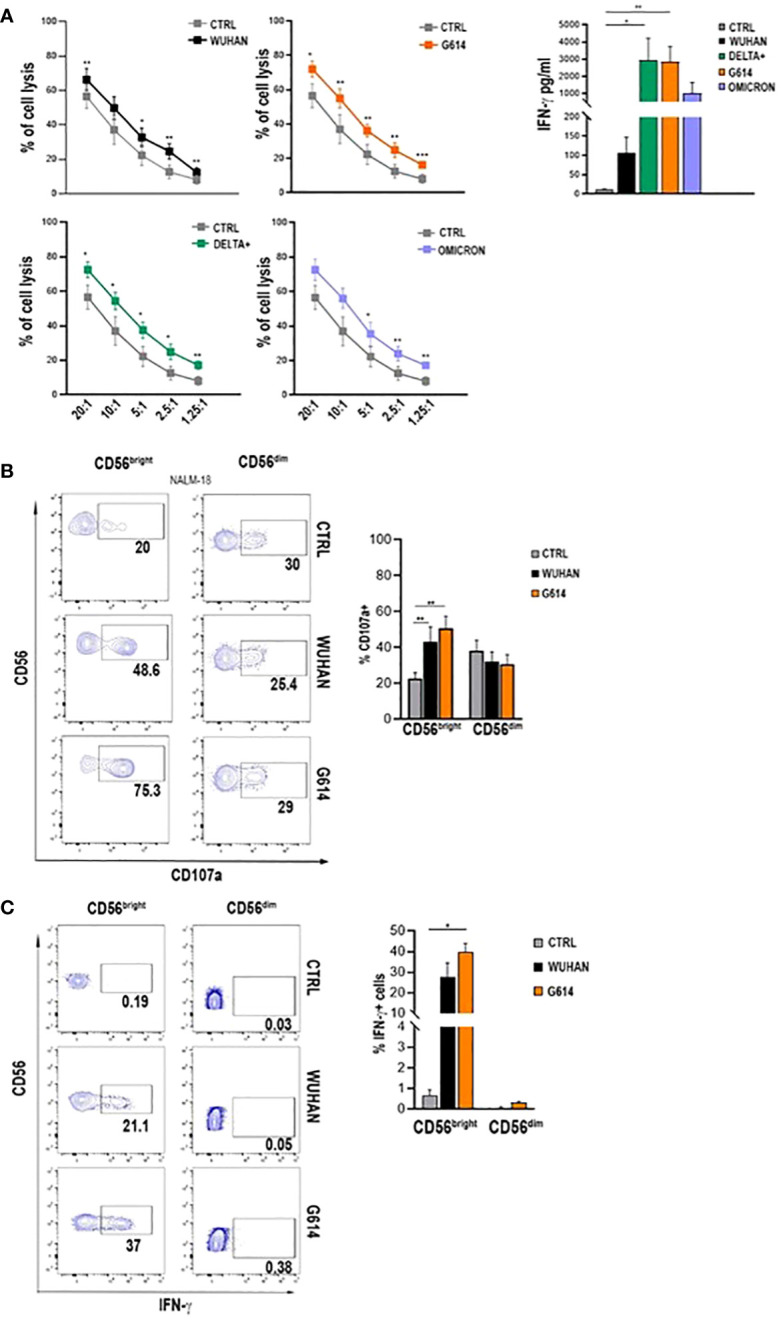
VOC-rSP improves NK cell function. **(A)** Cytotoxic activity (left panels) and IFN-γ production (right panel) (pg/mL) of VOC-treated (colored symbols as indicated) or untreated (CTRL, gray symbols) NK cells following co-culture with the NALM-18 cell line at the indicated effector (E):target (T) ratios. Data are plotted as percent of cell lysis of seven independent experiments performed. **(B)** CD107a expression by rSP (Wuhan and G614)-stimulated CD56^bright^ and CD56^dim^ NK cells co-cultured for 4h with NALM-18 cells (E:T = 1:1). Data of one representative experiment (left panels) and mean values of percentages of CD107a expression ( ± SEM) of seven experiments (right panels) are reported. **(C)** Representative dot plots (left panels) and the mean values of four experiments (right panel) of intracellular interferon-γ (IFN-γ) production by rSP (Wuhan and G614)-stimulated CD56^bright^ and CD56^dim^ NK cells. *p < 0.05, **p < 0.01, ***p <0.001.

All VOC-rSPs induced a statistically significant increase in the frequency of cells expressing the activation markers CD25 and CD69 but not of HLA-DR (not shown) as compared to untreated NK cells. Of note, upon treatment with G614, Delta-plus, and Omicron, higher proportions of NK cells expressing activation markers were observed as compared to the Wuhan strain. This effect was more evident in the CD56^bright^ than in the CD56^dim^ NK cell subset (**Figure 1D**; **Supplementary Figure 1C** and data not shown). Of note, rSPs from VOC-G614 and Delta-plus induced activation of NK cells in the great majority of HD assayed (**Supplementary Table 3**). In agreement with the above data, VOC-rSP-stimulated NK cells exerted significantly higher cytotoxic activity (left panels) and IFN-γ release (right panel) as compared to untreated NK cells upon co-culturing with an NK-sensitive cell line (NALM-18) (**Figure 2A**). Notably, rSP from both G614 and Delta-plus strains determined a significant increase of the IFN-γ release compared to the Wuhan strain (**Figure 2A**, right panel). Among NK cell subsets, VOC-rSP-stimulated CD56^bright^ NK cells displayed higher CD107a and IFN-γ expression compared to CD56^dim^ NK cells (**Figures 2B, C**).

### VOC-rSPs activate NK cells through TLR2 and TLR4 engagement

To establish which receptors are involved in rSP-NK cell binding and activation, we initially focused on ACE2 and the main NK cell activating receptors. NK cells did not express ACE2 at different time points of rSP stimulation (**Supplementary Figure 2C**). In addition, the masking experiments with neutralizing anti-ACE2 monoclonal antibody (mAb) did not affect CD25 and CD69 expression on NK cells induced by VOC (Wuhan, Delta-plus, G614)-rSP treatment (**Supplementary Figure 2D**). CD25/CD69 expression, as well as IFN-γ production on rSP-stimulated NK cells, was not inhibited by NKp46, NKp30, and NKp44 (NCR) and LFA1 neutralizing mAbs (**Supplementary Figures 3A, B**). Likewise, the expression on NK cell subsets of NKp46, NKp30, NKp44, LFA1, DNAM-1, and NKG2D was not modified by activation with VOC-rSPs (data not shown).

**Figure 3:**
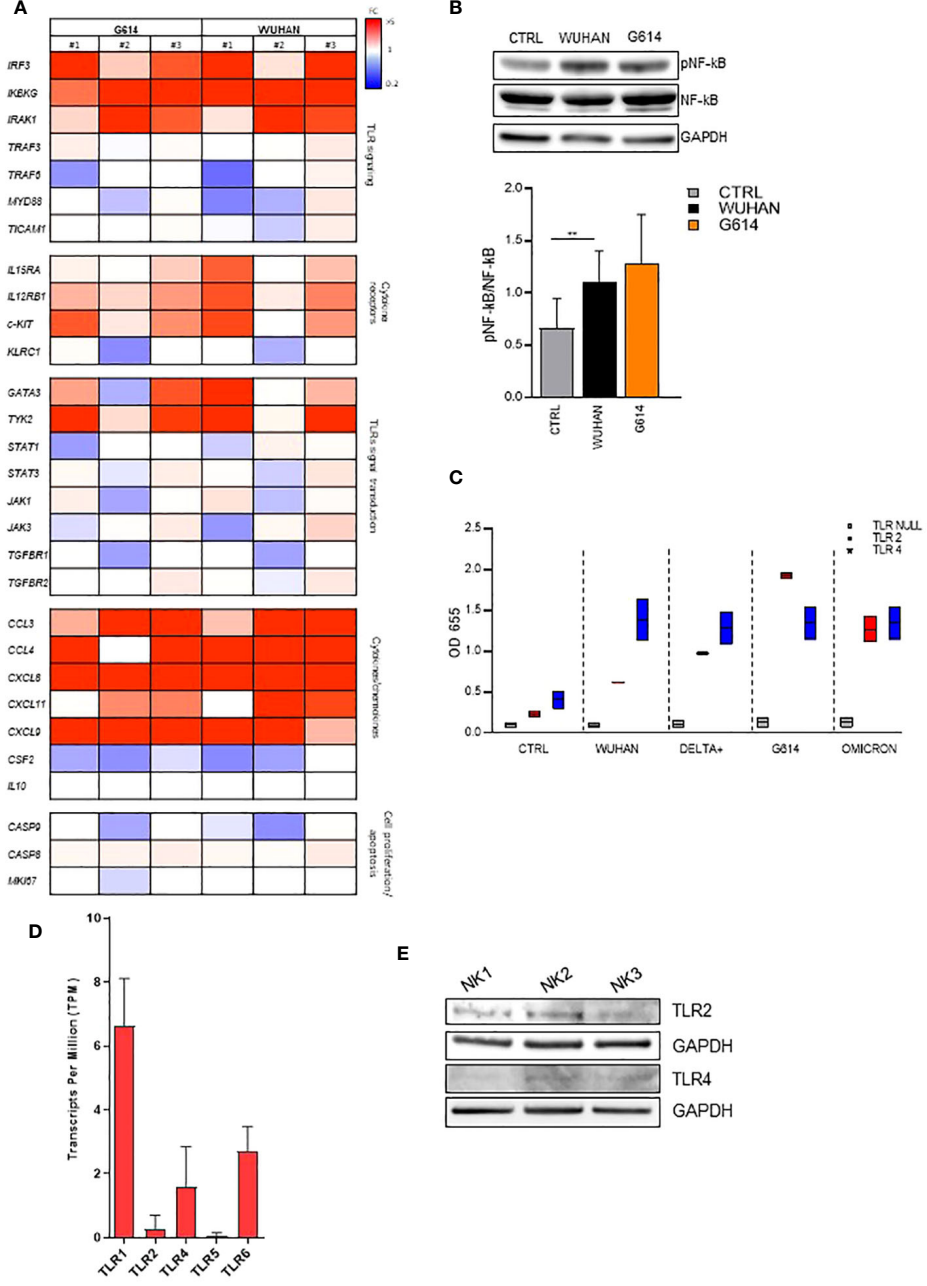
VOC-rSP binds and activates the TLR-signaling pathway. **(A)** Gene-expression heatmaps represented as a ratio between VOC (G614 and Wuhan)-stimulated and non-stimulated NK cells. Gene expression profiles are analyzed using 384-well TaqMan array microfluidic cards. mRNA intensities are displayed as colors ranging from red to blue as shown in the key. The reported genes are divided into distinct categories according to their function. The heatmap for VOC-rSP treatment conditions is relative to three experiments (FC = fold change). **(B)** Western Blot analysis and quantification of phosphorylated and total NF-kB of VOC (Wuhan and G614) rSP-treated and untreated (CTRL) NK cells. A representative blot (upper panel) from one out of three experiments (lower panel) is reported. Results have been normalized over GAPDH. **p < 0.01. **(C)** Binding of VOC- Wuhan, Delta-Plus, G614, Omicron) rSPs to TLR2- or TLR4-transfected (or non-transfected, TLR Null) HEK293 cell lines. Data shown are relative to two independent experiments. **(D)** Mean values of TLR1, TLR2, TLR4, TLR5, and TLR6 mRNA expression on bulk NK cells from 3 HD. **(E)** Western blot analysis of TLR2 and TLR4 on freshly isolated NK cells from three HD. Results have been normalized over GAPDH.

To further investigate the nature of the NK molecule(s) engaged by rSP, we evaluated the gene activation and the phosphorylation of some transcriptional factors upon rSP stimulation. The results of a customized CARD gene array performed on NK cells from PBMCs of three HD shortly (6 h) cultured with VOC (G614 and Wuhan)-rSPs indicated that several genes encoding for NK cell receptors, chemokines, signal transduction, transcription factors, and apoptosis were upregulated in stimulated compared to non-stimulated NK cells (**Figure 3A**). In particular, IRAK1, IRF3, and IKBKG genes (intermediate and terminal factors of the surface TLR signaling cascade) were highly upregulated (**Figure 3A**). Moreover, the same rSPs were able to induce the phosphorylation of NF-kB, strengthening the hypothesis of the possible role of surface TLR engagement (**Figure 3B**). Since *in silico* and structural studies indicated the direct interaction of rSP with TLR2 and TLR4 (30, 31), we checked the direct binding of all VOC-rSPs to TLR2 or TLR4 stable-transfected HEK-293 cell lines. rSPs from all VOCs bound both TLR2 and TLR4 with different binding capacity: Wuhan and Delta-plus rSPs bound TLR4 more than TLR2, while the inverse occurred for G614-rSP (**Figure 3C**).

On freshly isolated NK cells, TLR2 and TLR4 were expressed at the mRNA (**Figure 3D**) and protein (Western blotting) (**Figure 3E**) levels. However, the cytofluorimetric analysis indicated that both TLRs were poorly detectable on CD56^bright^ and CD56^dim^ NK cells and their expression was unchanged after rSP stimulation (**Supplementary Figures 4A, B**).

**Figure 4:**
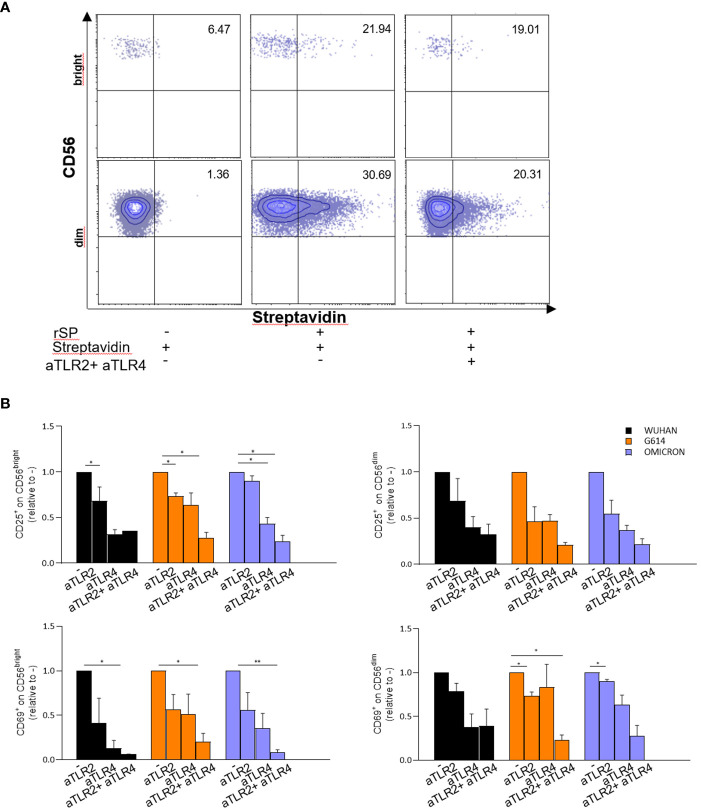
rSP activates NK cells through TL R2 and TLR4 engagement. **(A)** Representative dot plots reporting percentages of binding of biotinylated rSP (Wuhan) to CD56^bright^ and CD56^dim^ NK cells in the absence or presence of anti-TLR2 plus anti-TLR4 neutralizing antibodies. **(B)** CD25 and CD69 expression on VOC (Wuhan, G614, Omicron) rSPs-stimulated CD56^bright^ and CD56^dim^ NK cells co-cultured with anti-TLR2 or anti-TLR4 neutralizing antibodies alone or in combination. Graphs show the fold change calculated from the percentage of anti-TLR2- or anti-TLR4-treated samples relative to untreated samples (CTRL) (*n* = 3). Statistical analysis has been performed using paired *t*-test. *p < 0.05; **p < 0.01.

**Figure 5:**
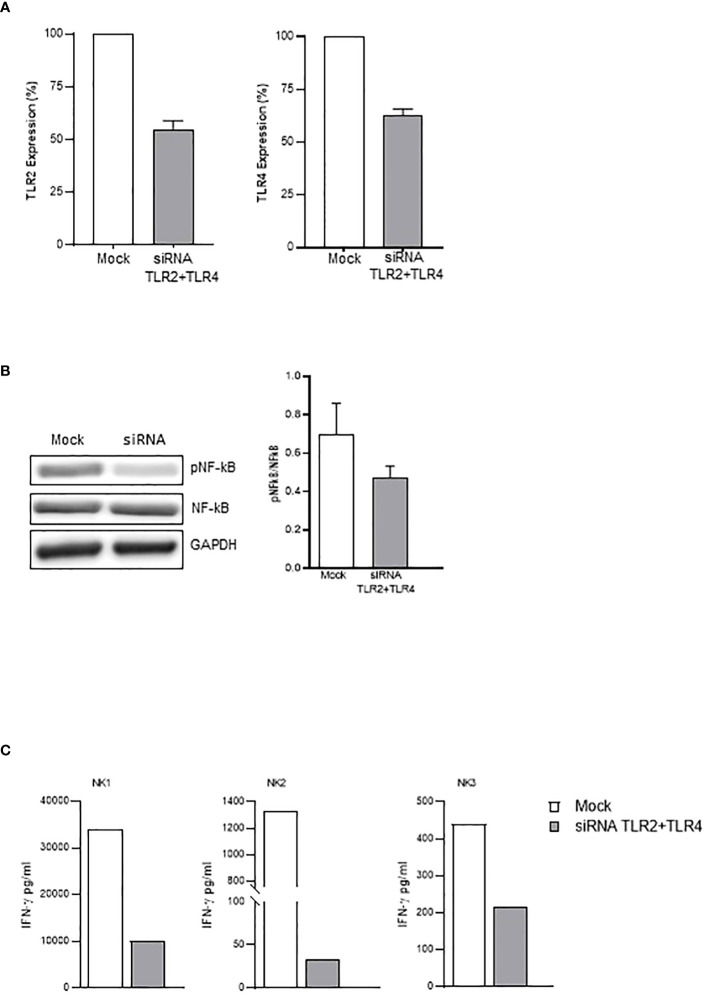
TLR2- and TLR4-silenced NK cells display reduced activation by rSP. **(A)** Percentage of inhibition of TLR2 (left) and TLR4 (right) mRNA expression on siRNA- vs. mock-transfected NK cells. The mean values ( ± SEM) of five experiments are reported. **(B)** Western blot analysis and quantification of phosphorylated and total NF-kB of mock-transfected and TLR2/TLR4-silenced NK cells following 24-h activation with rSP (Wuhan). A representative blot from one out of four independent experiments and quantification is reported. Results have been normalized over GAPDH. **(C)** IFN-γ production (pg/mL) of mock-transfected and TLR2/TLR4-silenced NK cells following 24-h activation with rSP (Wuhan) of 3 independent experiments.

In agreement with these data, subsequent experiments demonstrated that neutralizing anti-TLR2 and -TLR4 antibodies inhibited the binding of biotinylated-SP (Wuhan strain) to freshly isolated NK cells (**Figure 4A**). More importantly, the combination of the two neutralizing antibodies resulted in the strongest inhibition of the CD25/CD69 expression compared to the partially and variably downregulation observed upon treatment with single neutralizing Abs (**Figure 4B**; **Supplementary Figure 4C**). In line with these data, a reduction of IFN-γ and TNF-α production (**Supplementary Figure 4D**) was observed. Notably, the amount of IFN-γ released by rSP-stimulated NK cells was similar, often higher, to that produced in the culture with both TLR ligands [LPS, TLR4-ligand and Pam (3)csK(4), TLR2-ligand], suggesting similar affinity for the receptor(s) between rSP and the two agonists (**Supplementary Figure 4E**). Lastly, the silencing of TLR2 and TLR4 genes in NK cells from HD, by the transient transfection with specific siRNAs, inhibited rSP-induced phosphorylation of NF-kB and IFN-γ production by more than 50% (**Figures 5A–C**).

### CD56^dim^ NK cells from recovered patients respond efficiently to VOC-rSP stimulation

**Figure 6:**
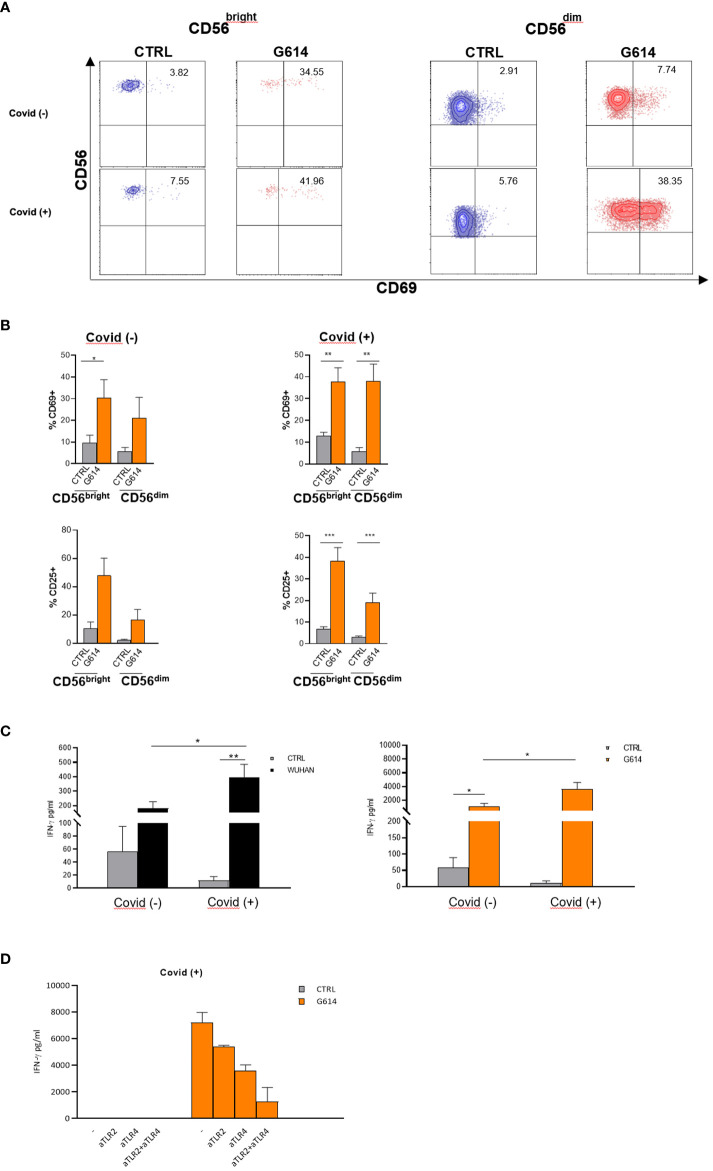
NK cells from recovered patients respond more efficiently to VOC-rSPs stimulation. **(A)** Dot plots reporting percentages of CD69 expression on rSP (G614)-stimulated CD56^bright^ and CD56^dim^ NK cells (gated from PBMC); data of one representative experiment out of 6 Covid− and 11 Covid+ patients. **(B)** The mean values ± SEM on rSP (G614)-stimulated CD56^bright^ and CD56^dim^ NK cells (gated from PBMC) of CD69 and CD25 of 6 Covid− and 11 Covid+ patients. Statistical analysis was performed using paired *t*-test *p < 0.05**p < 0.01***p < 0.001. **(C)** IFN-γ production (pg/mL) by VOC (Wuhan, G614)-stimulated PBMC. Statistical analysis has been performed (Covid− *n* = 5, Covid+ *n* = 11) using paired *t*-test *p < 0.05; **p < 0.01. **(D)** IFN-γ production (pg/mL) of PBMC from two recovered subjects (Covid+), pretreated with anti-TLR2-, anti-TLR4- neutralizing antibodies alone or in combination.

In order to evaluate the possible role of activated NK cells during SARS-CoV-2 infection, we examined the CD25 and CD69 expression and IFN-γ production of VOC (Wuhan and G614)-rSP-stimulated PBMCs from recovered patients (Covid+) and non-infected (Covid−) individuals who, at the sampling time, had received three doses of mRNA vaccine and were SARS-CoV-2 antigen-negative. We analyzed NK cells (gated as CD3^-^CD56^+^) among VOC-rSP-stimulated PBMCs from Covid− subjects and observed higher proportions of CD25 and CD69 expression almost exclusively in the CD56^bright^ NK cell subset than in not stimulated cells (**Figure 6B**). By contrast, significantly higher proportions of both CD56^bright^ and CD56^dim^ NK cells expressing CD69 activation marker were found in Covid+ patients (**Figure 6A**). Accordingly, the IFN-γ release was significantly higher in culture supernatants of VOC (Wuhan and G614)-rSP-stimulated PBMCs from the Covid+ group as compared to the Covid− group (**Figure 6C**). Notably, also in this set of experiments, the highest activation of NK cells was observed upon G614-rSP stimulation compared to other rSPs (data not shown). Lastly, IFN-γ production was inhibited *in vitro* by anti-TLR2 plus anti-TLR4 neutralizing Abs (**Figure 6D**).

On the whole, the results on cytokine production and on the expression of activation markers show that the proportion of rSPs *in vitro* activable NK cells is highly increased during infection and after recovery, prevalently involving the CD56^dim^ subset.

## Discussion

This study provides evidence that SP, a crucial component of SARS-CoV-2, binds to NK cells through novel receptors, namely, TLR2 and TLR4. Their engagement triggers cell activation leading to induction of cytotoxicity and cytokine production. These data indicate a direct effect of the virus on NK cells and imply a novel role of these cells in the pathogenesis of the disease.

In severe COVID-19 patients, NK cells are dysfunctional, express inhibitory receptors (as CD94/NKG2A), and display an exhaustion profile (13, 15). Many reports suggested that NK cell alterations are due to mechanisms that do not involve direct virus–NK cell interaction but, rather, to signals from cells of the inflammatory milieu (1, 12, 32, 33). Decrease in NK cell numbers may reflect a redistribution from blood to tissue. However, the finding of few mature NK cells in BALF is in contrast to their redistribution to the lung (14, 34). Recognition through CD94/NKG2A of an S1-derived HLA-E-binding peptide expressed by HLA-E+-infected epithelial cells may favor inhibition of NK cell function (35). NK cell number decrease and functional impairment are associated with a deficit of NK-stimulating cytokines (as IL-12 and IL-15) (<>2) released from infected and non-infected APCs, which, instead, produce suppressor molecules (IL-10 and TGF-β). Fibroblasts and epithelial and endothelial cells also release TGF-β upon virus stimulation, contributing to NK cell dysfunction (10). The overproduction of IL-6 inhibits NK cell cytotoxicity (36, 37), as confirmed by the *in vivo* treatment with therapeutic anti-IL-6R mAb, which increases NK cell function in COVID-19 patients (38). An extensive and comprehensive review on the causes of NK cell hyperactivation and dysfunction in COVID-19 has been recently reported (39).

Despite the fact that described mechanisms may lead to an impairment of NK cell number and function along the course of the disease, no data are available so far on the possibility of a direct effect of the SARS-CoV-2 or its rSP on NK cells, as well as of many other viruses or gps of the envelope (21). The present study aimed to establish whether rSP could directly act on NK cells.

The novel message of this study is that VOC-rSPs can directly bind and activate *in vitro* resting NK cells from HD, enrolled irrespective of previous infection or vaccination. SP subunit S1 induced higher NK cell stimulation than S2, whereas among SARS-CoV-2 gps, E, but not M and N gps, slightly activated the CD56^bright^ NK cell subset. In addition to the expression of activation markers (CD25 and CD69), different VOC-rSPs increased both cytokine release and cytolytic activity of freshly isolated NK cells. Although not shown, rSP-stimulated NK cells neither modulate LAG3, TIGIT, and PD1 genes nor determine any increase of CD57 or Annexin V surface expression. By contrast, NKG2C expression was higher in SP-stimulated than in untreated NK cells, likely mimicking the non-specific expansion of the “adaptive” NK cell subset and suggesting the induction of an activated rather than an exhausted profile.

Of note, only one report indicated the direct activation of NK cells with two SP peptides through their binding to NKG2D (40). The presence of free S1 protein is a relatively frequent finding in a SARS-CoV-2 inflamed environment due to its release from virus-infected and apoptotic cells. This result highlights the importance of our findings and strongly suggest the involvement of soluble S1 in the pathophysiology of the disease. High plasma levels of S1 have been reported during severe disease, PASC, and post-vaccination side effects (17–19, 41), indicating that NK cells can potentially be stimulated *in vivo* throughout the entire course of infection. Lastly, the SP stimulation of NK cells *in vivo* can also explain the increased plasma and tissue levels of IFN-γ described as a very early event of infection (12) and initially attributed exclusively to virus-specific CD8^+^ T cells.

Another novel and important information of this study concerns the activity of different VOC-rSPs on NK cells. Indeed, some VOC (G614, Delta-Plus and Omicron)-rSPs are more active in stimulating NK cells than others (Wuhan, Delta, and Epsilon). Indeed, the former rSPs activate NK cells at lower doses and usually induce higher CD25/CD69 expression and cytokine production by NK cells than Wuhan-rSP in most HD (unpublished). Intriguingly, the most active VOC-rSPs on NK cells are also those that displayed the greatest infectivity on the general population during the pandemic (42).

To delve into the mechanisms through which SP directly stimulates NK cells, in this study, we focused on the receptors engaged by different VOC-rSPs in NK cells. We found that NK cells did not constitutively display ACE2 receptor and that neither ACE2 nor the expression of activating receptors, including NCRs, LFA-1, DNAM-1, and NKG2D, changed significantly upon rSP stimulation. More importantly, mAbs neutralizing such receptors did not inhibit activation marker expression and cytokine production driven by VOC-rSPs on NK cells.

Further experiments revealed that rSP induced the activation of IRAK1, IRF3, and IKBKG genes and of the NF-κB pathway in NK cells, suggesting the involvement of one or more TLRs. This notion is not new since it is known that NK cells express functional TLRs involved in the recognition of pathogen-associated molecular patterns from Herpes-viridiae and bind the majority of gps from such virus family through TLR2 or, partially, TLR4 (21–24, 43).

By using TLR-transfected HEK-293 cell lines, we could clearly show that VOC-rSPs bound to both TLR2 and TLR4 with different binding capacities. Thus, Wuhan and Delta-Plus rSPs bound TLR4 more than TLR2, while the opposite occurred for G614-rSP. Of note, the combination of neutralizing TLR4 and TLR2 mAbs could strongly inhibit the SP-binding to both transfected HEK293 and NK cells as well as the CD25/CD69 upregulation and the cytokine production induced by VOC-rSPs on these cells. The engagement of the two receptors by rSP is indirectly confirmed by the finding that NK cells co-cultured with both TLR2 and TLR4 ligands produced a similar amount of IFN-γ than that of rSP stimulation, suggesting similar affinity of both stimuli for such TLRs. A definitive confirmation that the engagement of the two receptors was given with TLR2- and TLR4-silenced NK cells showed a consistent downregulation of rSP-driven phosphorylation of NF-kB and IFN-γ production when stimulated with rSP.

Notably, in agreement with these data, the heat-killed Bacillus Calmette-Guerin and Brassica Rapa L, containing sequences binding both TLR2 and TLR4, have been shown to directly activate NK cells *in vitro* or *in vivo*, respectively (44–46).

An important issue concerns the discrepancy between high rSP activity on NK cells and the low expression of both TLR receptors evaluated by flow cytometry. Indeed, both receptors are detectable at mRNA and protein levels but are poorly expressed at the cell surface. This is likely due to the unreliability of commercial-specific antibodies and also the effect of other mechanisms (24, 47–51). Several reports have clearly shown that some specific TLR2 or TLR4 ligands directly activate NK cells even though they display a barely detectable expression of the two receptors, thus suggesting that few surface molecules can still maintain their signaling ability when triggered by specific agonists (24, 52–54).

The interplay between SP and TLRs is complex and it is crucial to clarify the immunopathology of COVID-19. Molecular docking studies showed a direct interaction between human TLR4 (and TLR2) with SP on monocytes/macrophages (30, 31, 55, 56), which induced proinflammatory responses in APCs (57). The silencing of TLR4 in monocytes and monocyte cell lines, as well as its molecular inhibition, clearly confirmed a tight S1–TLR4 interaction (58, 59). Whether the engagement of TLR2 and TLR4, besides the important effect on NK cell activation and proinflammatory cytokine production, may also function as an entry receptor for the virus leading to NK cell infection, is still unknown. Taken together, these data suggest that the interaction of rSP with TLR2 and TLR4 on NK cells may have a strong impact on the immunopathology of COVID-19 and provides a clue that such receptors and/or their intracellular pathways could represent potential therapeutic targets.

The role of SP-activated NK cells during SARS-CoV-2 infection and disease progression needs to be better elucidated. It is likely that a difference in NK cell activation may exist, reflecting different VOCs, the status of *in vivo* NK cells, and/or possible TLR polymorphisms (60, 61). The finding that recently recovered subjects show an increased proportion of NK cells that can be activated *in vitro* by rSP (high CD25/CD69 expression on CD56^dim^ subset and high IFN-γ release) could explain why NK cells (mainly CD56^dim^ subset) are highly activated *in vivo* during infection and recovery. These data are also in agreement with the results of our study indicating that rSPs *in vitro* amplify the NKG2C^+^CD56^dim^ NK cell subset. Additional experiments, including HLA-E/peptide recognition, are needed to evaluate if this may be the result of trained immunity maintained over time. In agreement with our data, some studies described a significant increase of circulating NK cells after the resolution of the infection (62, 63), while others observed that increased adaptive NKG2C^+^CD57^+^NK cells from recovered subjects can mount a specific immune response against soluble SP peptides by secreting IFN-γ (64). In any case, whether NK cells from recovered subjects are protective during re-infections remains to be elucidated.

In conclusion, this report provides evidence that SARS-CoV-2 rSP, as the majority of gps of many viruses, directly engages both TLR2 and TLR4 molecules on NK cells and triggers intracellular pathways inducing their activation and function. This mechanism may allow to dampen viral infection at early stages, while contributing to excessive inflammation at late stages. Our present data strongly suggest an important role of NK cells in orchestrating the pathophysiology of different phases of COVID-19 and of some post-vaccination side effects.

## Data availability statement

The original contributions presented in the study are included in the article/**Supplementary Material**. Further inquiries can be directed to the corresponding author.

## Ethics statement

The studies involving humans were approved by Ethical Committee of IRCCS Bambino Gesù Children’s Hospital (2058_OPBG_2020). The studies were conducted in accordance with the local legislation and institutional requirements. The participants provided their written informed consent to participate in this study.

## Author contributions

NL: Writing – review & editing, Writing – original draft. BR: Writing – review & editing, Writing – original draft. IV: Writing – review & editing, Writing – original draft. CA: Writing – review & editing. FM: Writing – review & editing. AP: Writing – review & editing. LQ: Writing – review & editing. PV: Writing – review & editing. EPM: Methodology, Writing – review & editing. RC: Writing – review & editing. NT: Writing – review & editing. BA: Writing – review & editing. LM: Writing – review & editing. EM: Writing – review & editing, Writing – original draft.
