# Supplementary material for: TLR2/4 are novel activating receptors for SARS-CoV-2 spike protein on NK cells
Source: Front Immunol. 2024 May 31;15:1368946. doi: 10.3389/fimmu.2024.1368946 (PMC11176535; doi:10.3389/fimmu.2024.1368946)
Supplement: Supplementary file 1 [file DataSheet_1.docx]

Supplementary Material

**Supplementary Figures**


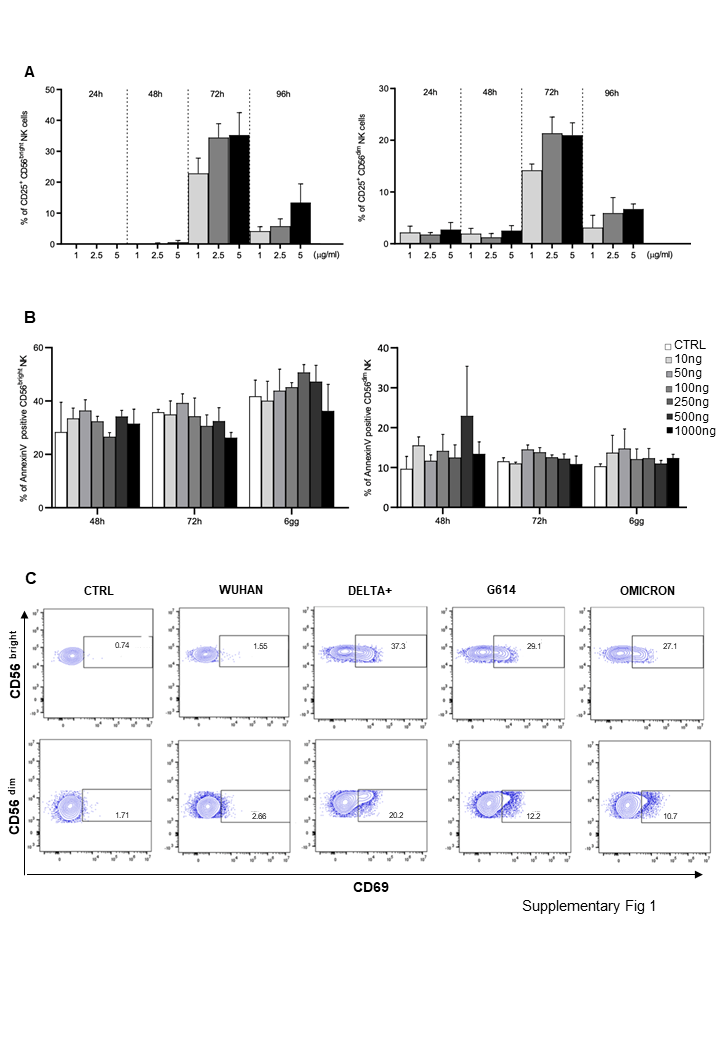


**Supplementary Figure 1. Optimization of experimental conditions for rSP stimulation of NK cells.** A. Time course experiments and dose-response curve of CD25 expression of (CD56^bright^ and CD56^dim^NK cell-gated) rSP (Wuhan)-stimulated PBMC (n=3). B. Dose-response curve of rSP (Wuhan) on the Annexin V expression by NK cell gated from PBMC (n=3). C. Representative dot plots reporting percentages of CD69+ CD56^bright^ and CD69+ CD56^dim^ NK cells stimulated with different VOCs.


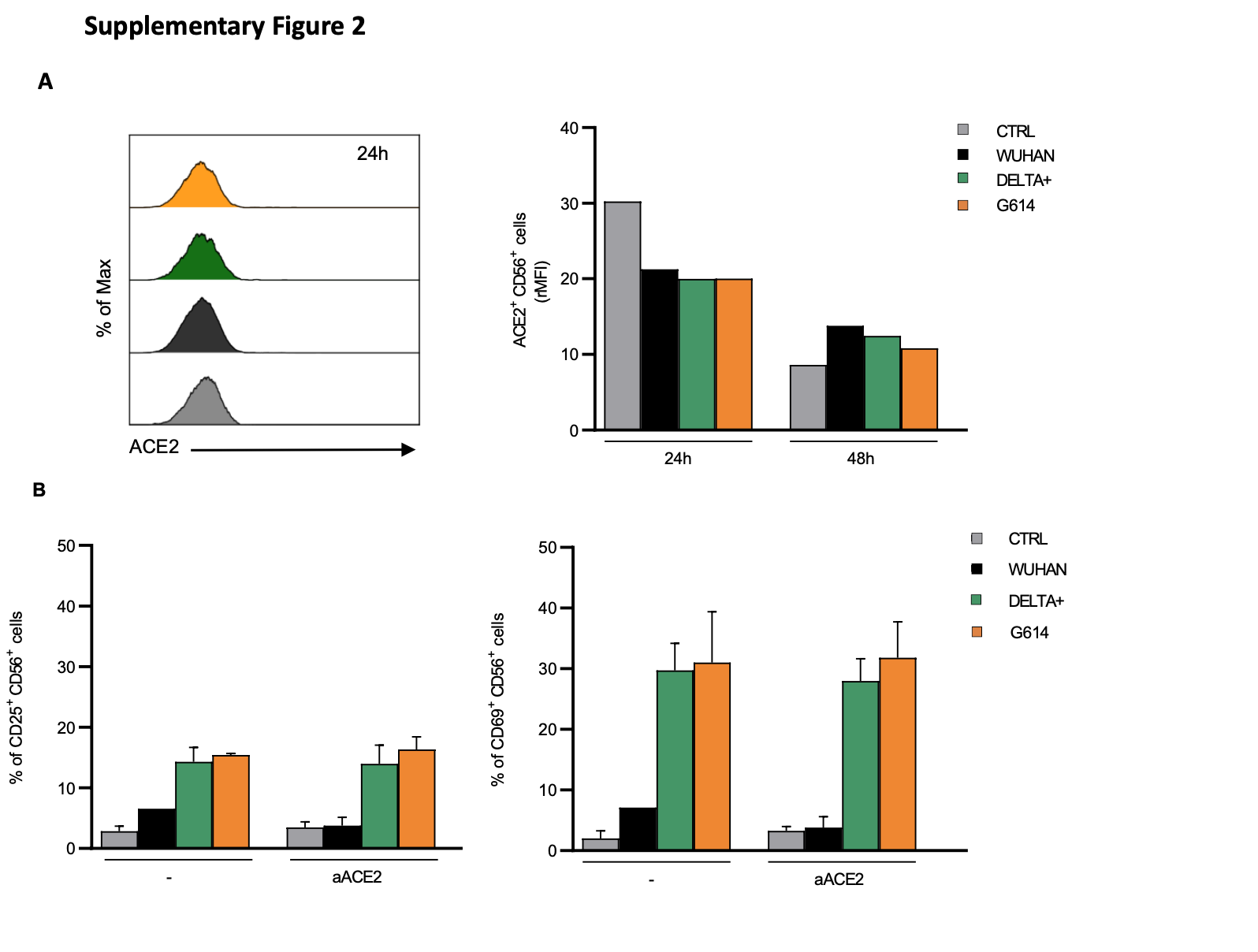


**C**

**D**

**B**

**A**

**CD56^bright^**

**CD56^dim^**

Supplementary Fig 2

**CTRL**

**CD56 ^dim^**

**CD56 ^bright^**

**CD25**

**M**

**E**

**N**

2.57

13.22

5.91

7.08

2.04

1.77

1.57

1.53

**Supplementary Figure 2. S1/S2 Wuhan subunits and ACE2 effects on NK cells.** A. Activity of recombinant S1 and S2 subunits of rSP (Wuhan) on NK cell subsets. Data are plotted as MFI (± SEM) of CD69 and CD25 expression of 3 experiments. B. One representative experiment of the activity of SARS-CoV2 E, M, and, N glycoproteins (2.5 µg/ml) on CD25 expression of CD56^bright^ and CD56^dim^ NK cell subsets. C. ACE2 expression evaluated at 24h and 48h on NK cells stimulated or not with VOC-(Wuhan, Delta plus, G614) rSPs. One representative experiment out of 4 performed is reported as percentage of max (left panel) and MFI (right panel) expression of labeled cells relative to unlabeled cells (rMFI). D. Percentages of CD25 and CD69 expression of VOC-(Wuhan, Delta Plus, and G614) rSP-stimulated NK cells treated with anti-ACE2 neutralizing antibody (n=2).

**
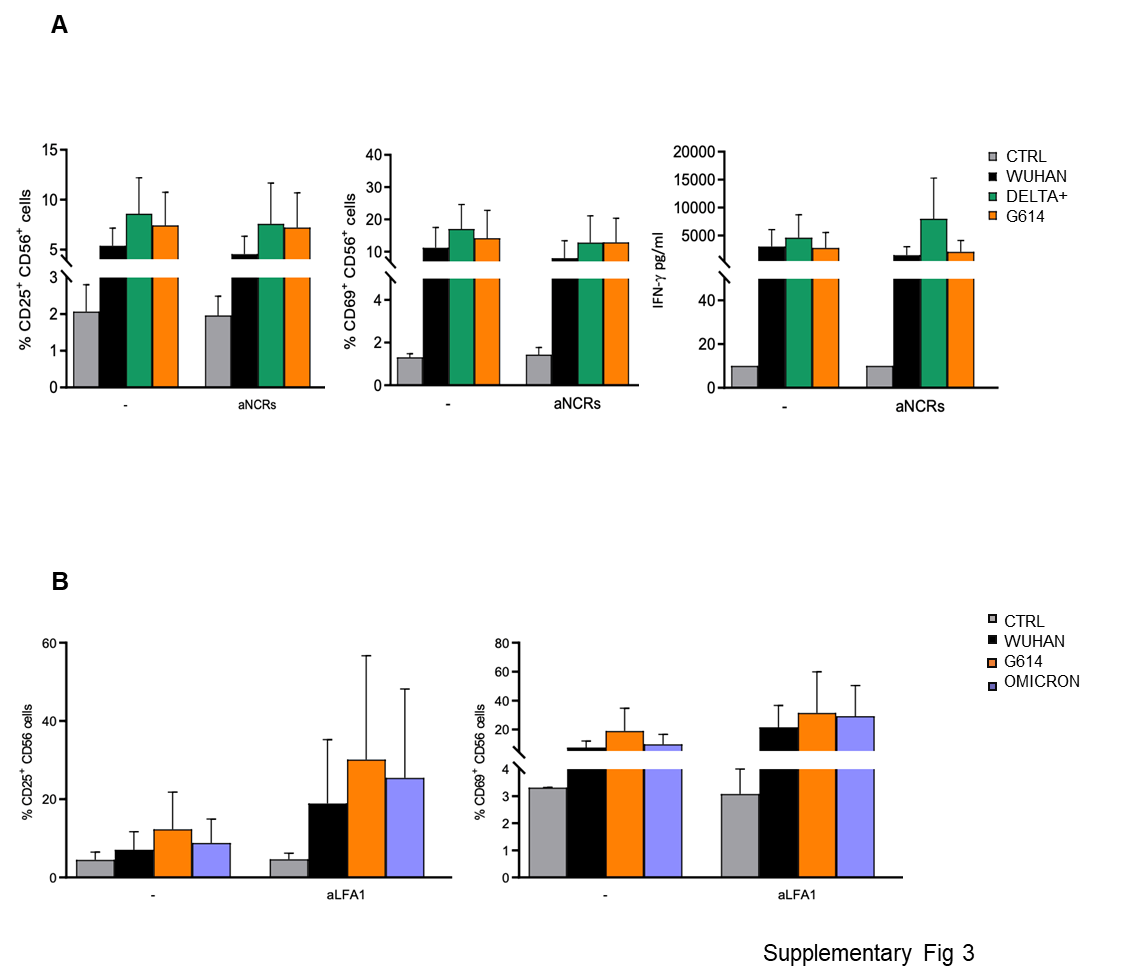
**

**Supplementary Figure 3. Effects of activating receptor masking on rSP-stimulated NK cells.** A. Percentages of CD25 and CD69 expression (left and middle panels) and IFN-γ production (pg/ml) on NK cells stimulated with VOC-rSPs in the absence or in the presence of neutralizing anti-NCR antibodies (n=3). B. Percentages of CD25 and CD69 expression on NK cells stimulated with VOC-rSPs in the absence or presence of neutralizing anti-LFA1 antibody (n=2).

**
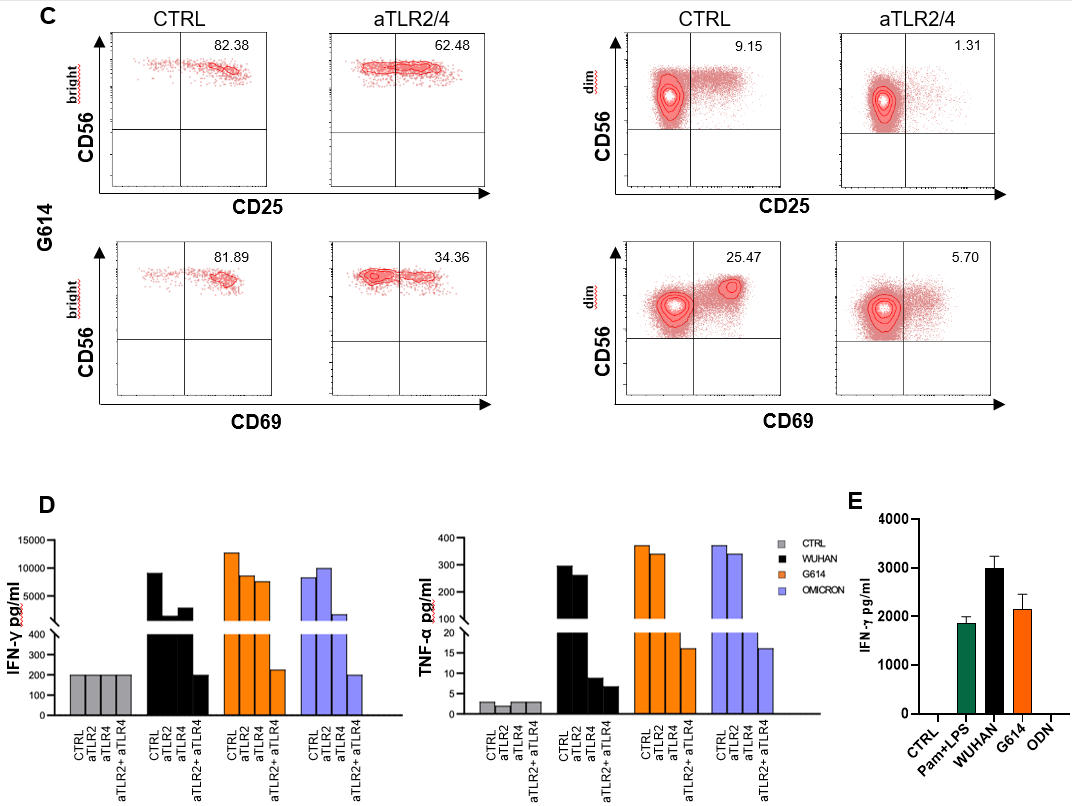

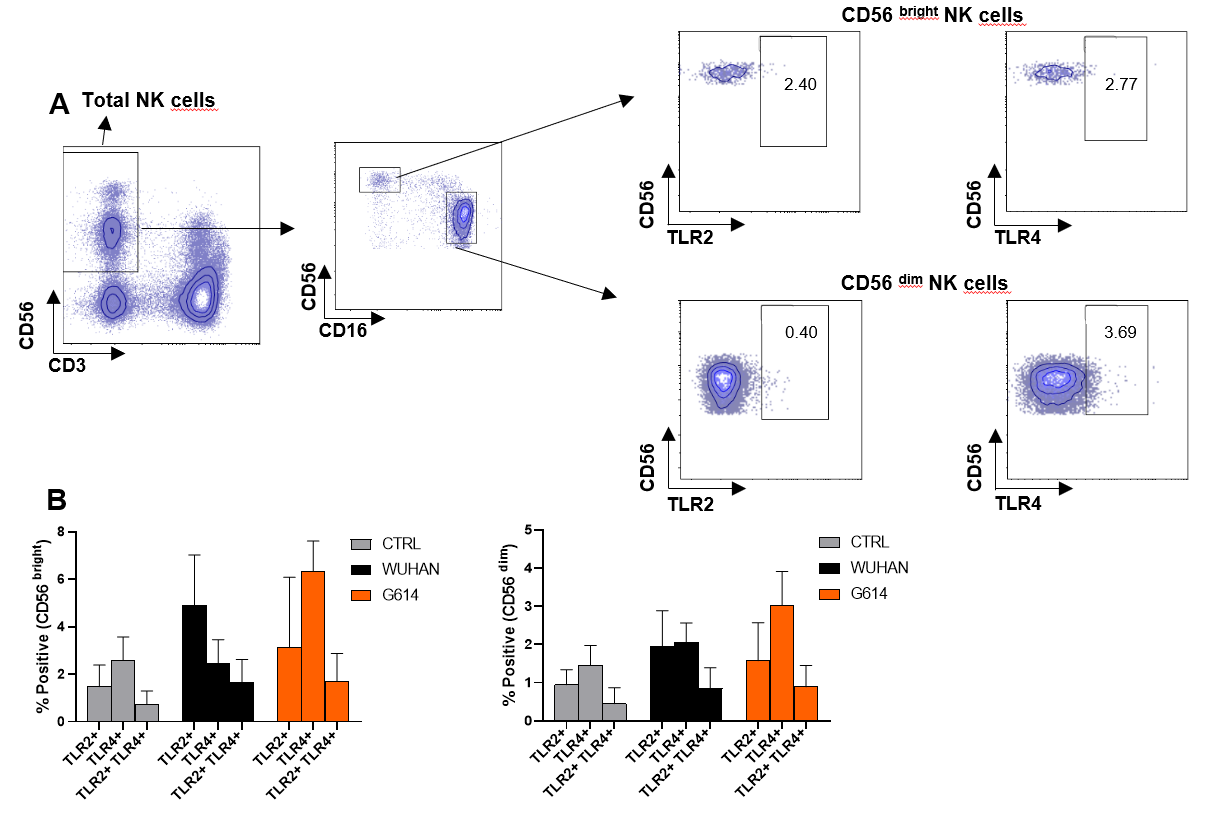
**

**Supplementary Figure 4. Expression of TLR2 and TLR4 and effects of TLR2/4 ligands and masking antibodies on rSP-stimulated NK cells.**

A. Gating strategy to detect TLR2 and TLR4 expression on NK cell subsets starting from PBMC. B. Percentages of TLR2+, TLR4+, and TLR2+TLR4+ gated on CD56^bright^ and CD56^dim^NK cells from PBMC of 7 HD stimulated with rSP (Wuhan and G614). C. Representative dot plots of CD25 and CD69 expression on rSP G614-stimulated CD56^bright^ and CD56^dim^ NK cells co-cultured in the absence or presence of anti-TLR2 and anti-TLR4 neutralizing antibodies. D. IFN-γ and TNF-α production (pg/ml) by VOC- (Wuhan, G614, Omicron) rSP-stimulated NK cells from one HD co-cultured with anti-TLR2 or anti-TLR4 antibody alone or in combination. E. IFN-γ (pg/ml) production by NK cells upon Pam(3)csK(4) (TLR2 agonist) plus LPS (TLR4 agonist)- vs rSPs (Wuhan, G614) and unrelated ODN 2243 stimulation (n=4).
