# Supplementary material for: TLR2/4 are novel activating receptors for SARS-CoV-2 spike protein on NK cells
Source: Front Immunol. 2024 May 31;15:1368946. doi: 10.3389/fimmu.2024.1368946 (PMC11176535; doi:10.3389/fimmu.2024.1368946)
Supplement: Supplementary file 2 [file Table_1.docx]

Supplementary Tables

Supplementary Table 1. Reagents’ list

| **REAGENT or RESOURCE** | **SOURCE** | **IDENTIFIER** |
| --- | --- | --- |
| ***Antibodies*** | | |
| **Anti-TLR2** | R&D | Cat #MAB2616 |
| **Anti-TLR4** | R&D | Cat # AF1478 |
| **Anti-integrin-b2** | R&D | Cat # AF1730 |
| **Anti-ACE2** | R&D | Cat # AF933 |
| **CD3-APC** | Imm.Sci | Cat # MAB-02APC |
| **CD3-ECD** | Beckman Coult | Cat # A07748 |
| **CD56-PC7** | Beckman Coult | Cat # A21692 |
| **CD56-BV650** | BD | Cat # 564057 |
| **CD57-PB** | Biolegend | Cat # 359608 |
| **CD16-BV510** | Biolegend | Cat # 302048 |
| **CD16-FITC** | BD | Cat # 555406 |
| **CD25-PE** | Miltenyi Biotech | Cat # 130-113-286 |
| **CD69-PerCP** | Becton Dickinson – BD- | Cat # 560738 |
| **CD107a (LAMP-1)** **eFluor 660** | Invitrogen | Cat # 50107942 |
| **TLR2-FITC** | eBioscience | Cat #11-9922-42 |
| **TLR4-APC** | eBioscience | Cat #17-9917-42 |
| **TLR4-APC** | Abcam | Cat # ab155343 |
| **IFN-γ-APC EF780** | eBioscience | Cat # 47-7319-42 |
| **Streptavidin BV421** | Biolegend | Cat # 405226 |
| **ACE2-APC** | R&D | Cat # FAB9332 |
| **Annexin V FITC** | BD | Cat # 556547 |
| **DNAM1** | Pende D. University of Genova | N/A |
| **NKp46** | Pende D. University of Genova | N/A |
| **NKp30** | Pende D. University of Genova | N/A |
| **NKp44** | Pende D. University of Genova | N/A |
| **NKG2A-PE-Cy7** | Beckman Coult | Cat # B10246 |
| **NKG2C-AF700** | R&D | FAB138N |
| **NKG2D** | Pende D. University of Genova | N/A |
| **Phospho- NF-kB** | Cell Signaling Technol | Cat # 3033s |
| **NF-kB** | Cell Signaling Technol | Cat # 8242s |
| **Anti-rabbit HRP** | Cell Signaling Technol | Cat # 7074P2 |
| **GAPDH** | Sigma Aldrich | Cat # G9545 100UL |
| **HLA-DR PerCP** | Miltenyi | Cat#130-113-404 |
| ***Biological samples*** |  |  |
| **Human Subjects Blood Samples** | Pediatric Hospital Bambino Gesù | N/A |
| ***Chemicals, peptides, and recombinant proteins*** | | |
| **Wuhan- Spike** | Proteogenix | Cat # PX-CoV-P049 |
| **Indian Delta Plus- Spike** | Proteogenix | Cat # PX-CoV-P062 |
| **Variant 3 G614- Spike** | Proteogenix | Cat # PX-CoV-P051 |
| **Omicron RBD- Spike** | Proteogenix | Cat # PX-CoV-P076 |
| **S1 subunit** | Proteogenix | Cat # PX-CoV-P002 |
| **S2 subunit** | Proteogenix | Cat # PX-CoV-P045 |
| **Envelope protein** | Proteogenix | Cat # PX-CoV-P077 |
| **Nucleoprotein** | Proteogenix | Cat # PX-CoV-P010 |
| **M protein** | Proteogenix | Cat # PX-CoV-P025 |
| **Biotinylated Spike** | Biotechne | Cat # BT-10549-050 |
| **GolgiStop Protein Transport Inhibitor (containing Monensin)** | BD | Cat# BD 554724 |
| **Lipopolysaccharide from Escherichia coli K12** | InvivoGen | Cat# tlrl-eklps |
| **ODN2243** | InvivoGen | Cat# tlrl-2243-1 |
| **Pam(3)csK(4)** | InvivoGen | Cat# tlrl-pms |
| **PBS 1X** | Capricorn scientific | Cat # pbs-1A |
| **Ficoll-Paque Plus** | Lympholite | Cat # CL5020 |
| **RPMI-1640** | Euroclone | Cat # ECM9106L |
| **DMEM** | Euroclone | Cat # ECM0102L |
| **2mM L-glutamine** | Euroclone | Cat # ECB3000D |
| **1% penicillin and streptomycin** | Euroclone | Cat # ECB3001D |
| **Fetal bovine serum FBS** | Gibco | Cat # 26140-079 |
| **Normocina** | Invivogen | Cat # ant-nr-1 |
| **Zeocina** | Invivogen | Cat # ant-zn-1 |
| **QUANTI-Blue Solution** | Invivogen | Cat # rep-qbs |
| **Propidium iodide** | Sigma-Aldrich | Cat # p4864-10 |
| **Cell Tracker Green** | Life Technologies | Cat # C2925 |
| **PVDF membrane** | Biorad | Cat # 162-0177 |
| **Not fat dry milk** | Cell Signaling Technology | Cat # 9999S |
| **Proteinase inhibitor** | Thermo Fisher Scientific | Cat # 78440 |
| **TRIS** | Sigma Aldrich | Cat # T6066 |
| **NP-40** | Thermo Fisher Scientific | Cat # 28324 |
| **Na-Deoxycholate** | Sigma Aldrich | Cat # D6750 |
| **NaCl** | Sigma Aldrich | Cat # S3014 |
| **EDTA** | Sigma Aldrich | Cat # E5134 |
| **PowerUp Sybr Green reagent** | Applied Biosystems | Cat # A25742 |
| **TaqMan Advanced Master Mix** | Applied Biosystems | Cat # 4444557 |
| **Critical Commercial assays** | | |
| **RosetteSep NK-cell enrichment cocktail** | STEMCELL Technologies | Cat # 15065 |
| **FoxP3 Staining Buffer Set** | Miltenyi Biotech | Cat # 130-093-142 |
| **DuoSet ELISA kits IFN-γ** | R&D | Cat # DY285b |
| **DuoSet ELISA kits TNF-α** | R&D | Cat # DY210 |
| **DuoSet ELISA kits CXCL10/IP-10** | R&D | Cat # DY266 |
| **DuoSet Ancillary Reagent Kit** | R&D | Cat # DY008 |
| **RNeasy Plus mini kit** | Quiagen | Cat # 74134 |
| **BCA protein assay kit** | Thermo-Fisher Scientific | Cat # 23225 |
| **ECL prime system** | Ge Healthcare | Cat # RPN2232 |
| **Super Script IV 1st-strand synthesis syst.** | Invitrogen | Cat # 18091050 |
| **384-well TaqMan array microfluidic card** | Thermo-Fisher Scientific | Cat # 4342259 |
| **Neon Transfection system** | Thermo-Fisher Scientific | Cat # mpk10096 |
| **Experimental models: Cell lines** | | |
| **NALM-18** | Cellosaurus | Cat # CVCL_5661 |
| **HEK-293 Blue Null1** | InvivoGen | Cat # hkb-null1 |
| **HEK-Blue hTLR2** | InvivoGen | Cat # hkb-htlr2 |
| **HEK-Blue hTLR4** | InvivoGen | Cat # hkb-htlr4 |
| **Oligonucleotides** | | |
| **Silencer select TLR2** | Thermo-Fisher Scientific | Assay ID: s168, s169 and s170 |
| **Silencer select TLR4** | Thermo-Fisher Scientific | Assay ID: s14194, s14195 and s14196 |
| **Negative control #2 siRNA** | Thermo-Fisher Scientific | N/A |
| **384-well TaqMan array microfluidic cards primers IDs** | Thermo-Fisher Scientific (Suppl Table 2) | N/A |
| **Software and algorithms** | | |
| **CytExpert 2.4** | Beckman Coulter |  |
| **FlowJo 10.9** | BD |  |
| **Kaluza 2.1** | Beckman Coulter |  |
| **Ninealliance© software** | Uvitec |  |
| **Prism V.9.3.1** | GraphPad |  |

**Supplementary Table 2. Selected genes analyzed with CARD gene array**

| **Target gene** | **ID gene** | **Target gene** | **ID gene** | **Target gene** | **ID gene** |
| --- | --- | --- | --- | --- | --- |
| **FCGR3A** | Hs02388314_m1 | **MAPK3** | Hs00385075_m1 | **TRAF6** | Hs00939742_g1 |
| **FCGR3B** | Hs04334165_m1 | **MAPK1** | Hs01046830_m1 | **TBK1** | Hs00179410_m1 |
| **NCAM1** | Hs00941830_m1 | **MAP2K1** | Hs00983247_g1 | **IRF3** | Hs01547283_m1 |
| **KLRC1** | Hs00970273_g1 | **EOMES** | Hs00172872_m1 | **IRF5** | Hs00158114_m1 |
| **KLRC2** | Hs02379574_g1 | **TBX21** | Hs00203436_m1 | **IRF7** | Hs01014809_g1 |
| **HAVCR2** | Hs00958618_m1 | **GATA3** | Hs00231122_m1 | **MYD88** | Hs01573837_g1 |
| **ITGB2** | Hs00164957_m1 | **FOXP3** | Hs01085834_m1 | **IRAK1** | Hs01018347_m1 |
| **TIGIT** | Hs00545087_m1 | **FOXO1** | Hs00231106_m1 | **IRAK4** | Hs00928779_m1 |
| **SIGIRR** | Hs00222347_m1 | **ID2** | Hs04187239_m1 | **NFKB1** | Hs00765730_m1 |
| **PDCD1** | Hs01550088_m1 | **ZEB2** | Hs00207691_m1 | **RELA** | Hs00153294_m1 |
| **GAPDH** | Hs99999905_m1 | **NOTCH1** | Hs01062014_m1 | **CHUK** | Hs00989497_m1 |
| **LILRB1** | Hs04401227_g1 | **TCF7** | Hs00175273_m1 | **IKBKB** | Hs01559460_m1 |
| **TGFBR1** | Hs00610320_m1 | **NFATC1** | Hs00542678_m1 | **IKBKG** | Hs00415849_m1 |
| **TGFBR2** | Hs00234253_m1 | **NFATC2** | Hs00905451_m1 | **CD40** | Hs00374176_m1 |
| **LAG3** | Hs00158563_m1 | **NFATC4** | Hs00190037_m1 | **CCL3** | Hs00234142_m1 |
| **KIT** | Hs00174029_m1 | **JUN** | Hs01103582_s1 | **CCL4** | Hs99999148_m1 |
| **ISG20** | Hs00158122_m1 | **SRF** | Hs00182371_m1 | **CXCL11** | Hs00171138_m1 |
| **IL12RB1** | Hs01106578_m1 | **TBP** | Hs00427620_m1 | **CXCL9** | Hs00171065_m1 |
| **IL15RA** | Hs00542602_g1 | **MAF** | Hs00193519_m1 | **18S** | Hs99999901_s1 |
| **IL2RG** | Hs00415671_m1 | **SPI1** | Hs02786711_m1 | **ACTB** | Hs99999903_m1 |
| **TYROBP** | Hs00182426_m1 | **USF1** | Hs00273038_m1 |  |  |
| **CD247** | Hs00609515_m1 | **GATA1** | Hs01085823_m1 |  |  |
| **HCST** | Hs00367159_m1 | **IL17A** | Hs00174383_m1 |  |  |
| **SYK** | Hs00895377_m1 | **CXCL8** | Hs00174103_m1 |  |  |
| **ZAP70** | Hs00277148_m1 | **IL10** | Hs00961622_m1 |  |  |
| **PTPN6** | Hs00169359_m1 | **CSF2** | Hs00929873_m1 |  |  |
| **JAK1** | Hs01026983_m1 | **CASP9** | Hs00154260_m1 |  |  |
| **JAK2** | Hs00234567_m1 | **CASP8** | Hs01018151_m1 |  |  |
| **JAK3** | Hs00354555_m1 | **BCL2** | Hs00608023_m1 |  |  |
| **TYK2** | Hs00177464_m1 | **FASLG** | Hs00181225_m1 |  |  |
| **STAT1** | Hs01013996_m1 | **MKI67** | Hs01032443_m1 |  |  |
| **STAT3** | Hs00374280_m1 | **TLR3** | Hs00152933_m1 |  |  |
| **STAT4** | Hs00231372_m1 | **TLR7** | Hs00152971_m1 |  |  |
| **STAT5A** | Hs00234181_m1 | **MAP3K7** | Hs00177373_m1 |  |  |
| **STAT5B** | Hs00560035_m1 | **TLR8** | Hs00152972_m1 |  |  |
| **SOCS1** | Hs00705164_s1 | **TLR9** | Hs00370913_s1 |  |  |
| **PIK3CA** | Hs00180679_m1 | **TICAM1** | Hs00706140_s1 |  |  |
| **MTOR** | Hs00234508_m1 | **TRAF3** | Hs00936781_m1 |  |  |

**Supplementary Table 3. Different VOCs activate NK cells of the majority of HD**

______________________________________________________________________________________

Percentages of HD* showing increased^§^ NK cells expressing

_________________________________________________________________

VOC-rSPs CD25+ CD69+ ________________________ __________________

CD56^Bright^ CD56^Dim^ CD56^Bright^ CD56^Dim^

__________________________________________________________________________________

Wuhan 60 28.5 49 31.4

Delta Plus 82.8 65.7 88.5 60

G614 91.4 62.8 94.2 82.8

Omicron 80 54.2 80 62.8

_______________________________________________________________________________________________________

* n = 35

^§^ At least twice the values of VOC-rSP-stimulated- vs untreated NK cells
